# Loss of *Elp1* perturbs histone H2A.Z and the Notch signaling pathway

**DOI:** 10.1242/bio.058979

**Published:** 2021-09-30

**Authors:** BreAnna Cameron, Elin Lehrmann, Tien Chih, Joseph Walters, Richard Buksch, Sara Snyder, Joy Goffena, Frances Lefcort, Kevin G. Becker, Lynn George

**Affiliations:** 1 Department of Biological and Physical Sciences, Montana State University Billings, Billings, MT 59101, USA; 2 Computational Biology & Genomics Core (CBGC), Laboratory of Genetics and Genomics (LGG), Department of Health and Human Services (DHHS), National Institute on Aging, Intramural Research Program (NIA IRP), National Institutes of Health (NIH), Biomedical Research Center, Baltimore, MD 21224, USA; 3 Department of Microbiology and Immunology, Montana State University, Bozeman, MT 59717, USA; 4 Gene Expression and Genomics Unit, National Institute on Aging, National Institutes of Health, Baltimore, MD 21224, USA

**Keywords:** Elongator, Elp1, H2A.Z, Histone, TSA, Notch

## Abstract

Elongator dysfunction is increasingly recognized as a contributor to multiple neurodevelopmental and neurodegenerative disorders including familial dysautonomia, intellectual disability, amyotrophic lateral sclerosis, and autism spectrum disorder. Although numerous cellular processes are perturbed in the context of Elongator loss, converging evidence from multiple studies has resolved Elongator's primary function in the cell to the modification of tRNA wobble uridines and the translational regulation of codon-biased genes. Here we characterize *H2a.z*, encoding the variant *H2a* histone H2A.Z, as an indirect Elongator target. We further show that canonical Notch signaling, a pathway directed by H2A.Z, is perturbed as a consequence of *Elp1* loss. Finally, we demonstrate that hyperacetylation of H2A.Z and other histones via exposure to the histone deacetylase inhibitor Trichostatin A during neurogenesis corrects the expression of *Notch3* and rescues the development of sensory neurons in embryos lacking the *Elp1* Elongator subunit.

## Abstract (precis)

**Summary:** The maldevelopment of sensory neurons in Elongator knockout embryos is associated with elevated H2A.Z and perturbed Notch signaling that can be rescued by Trichostatin A.

## INTRODUCTION

Familial dysautonomia (FD) is both a neurodevelopmental and neurodegenerative disease that ravages the peripheral nervous system (PNS). FD results from mutations in *ELP1*, a gene that encodes the scaffolding subunit for a six-subunit molecular complex called Elongator that is abundant in the cytoplasm of neurons. Studies characterizing a multitude of phenotypes among Elongator deletion models led to the hypothesis that this complex plays multiple cellular roles. However, an increasing body of evidence indicates that Elongator performs a single task; the addition of methoxy-carbonyl-methyl (mcm^5^) to tRNA U_34_ (Dauden et al., 2019; Lin et al., 2019; Selvadurai et al., 2014; Huang et al., 2005; Glatt et al., 2012, 2016). This modification and the subsequent mcm^5^-dependent thiolation (s^2^) of U_34_ increase the translational efficiency of AA-ending codons and decrease the translational efficiency of AG-ending codons for the amino acids lysine, glutamine, and glutamic acid (Nedialkova and Leidel, 2015; Agris et al., 1973; Bjork, 1995; Murphy et al., 2004; Suzuki, 2005; Yarian et al., 2000; Lustig et al., 1981; Sekiya et al., 1969; Yokoyama et al., 1985; Zinshteyn and Gilbert, 2013; Goffena et al., 2018). Through the addition of mcm^5^s^2^, Elongator thus fine tunes ribosomal elongation speed and hence the amount of protein produced from a given transcript, particularly those that exhibit a biased usage of either AA or AG-ending codons. As such, loss of Elongator impacts the levels of hundreds of proteins in the cell (Goffena et al., 2018), supporting the current paradigm wherein Elongator plays a single primary role (tRNA modification), that when lost results in a gamut of diverse downstream phenotypic effects.

FD children exhibit PNS impairments at birth. This, in combination with early autopsy studies showing a severely reduced number of neurons in both the sympathetic and dorsal root ganglia (DRG), underscores the developmental aspect of the disease (Pearson et al., 1978). To investigate Elongator's function in embryonic development, we previously generated a conditional knockout (CKO) mouse in which *Elp1* and hence Elongator function are deleted in the PNS. Unlike full *Elp1* knockouts that are early embryonic lethal, *Wnt1-Cre*; *Elp1^LoxP^*^/*LoxP*^ (*Elp1* CKO) pups are born alive. Furthermore, they exhibit reduced numbers of DRG and sympathetic neurons, with small diameter DRG neurons expressing the high affinity nerve growth factor receptor TrkA most severely impacted, recapitulating a hallmark FD phenotype (George et al., 2013; Pearson et al., 1978). We previously showed that this preferential loss of TrkA+ neurons is at least in part due to a loss of mitotically active progenitor cells (George et al., 2013). During normal development, the majority of TrkA+ neurons are born relatively late, during a second wave of neurogenesis that requires their progenitor cells to postpone both differentiation and proliferation until approximately E12 (Farinas et al., 1996, 1998; Lawson and Biscoe, 1979). We previously showed that this developmental pathway is compromised in *Elp1* CKOs, with some progenitors for TrkA+ neurons differentiating precociously, and others dying via p53 mediated apoptosis (George et al., 2013; Goffena et al., 2018). Without a supply of progenitors to mediate the second wave, the number of TrkA+ neurons in these embryos is significantly depleted at E12.5 and remains reduced throughout the duration of embryonic development (George et al., 2013).

To gain insight into the cellular and molecular mechanisms that precipitate the maldevelopment of the TrkA+ neuron population, we performed a combined transcriptome and proteome study to identify Elongator targets that exhibit altered expression in the context of compromised Elongator function (Goffena et al., 2018). In this previous study we identified two classes of genes: large AA-biased genes that exhibit reduced expression, and small AG-biased genes that exhibit elevated expression (Goffena et al., 2018). In this second category, we identified *H2a* histones as a family of functionally related genes whose expression levels are likely coordinated by Elongator via similarly structured transcripts including an extreme AG-bias. Here we show that the variant histone, *H2a.z*, is also an Elongator target, although likely via an indirect route, and that *H2a.z* protein levels are elevated in the context of *Elp1* ablation in multiple nervous system components and model systems. We go on to show that Notch signaling, a pathway regulated by H2A.Z, is perturbed in the context of *Elp1* loss and that hyperacetylation of H2A.Z (and other histones) *in vivo* corrects the expression of *Notch3* and rescues the development of sensory neurons in *Elp1* CKO mice.

## RESULTS

### H2A.Z levels are elevated in the absence of *Elp1* in multiple cell types and knockout models

In our previous transcriptome and combined proteome study of DRG from E17.5 embryos, we identified the canonical histone *H2a* genes as family of functionally related genes that are likely regulated by Elongator at the level of translation (Goffena et al., 2018). All canonical *H2a* genes encode small transcripts, consisting of only 129–131 total codons, and exhibit an extreme preference for AG-ending codons with an average AA:AG ratio of 0.08 in mouse (average AA:AG ratio across all open reading frames=0.67) (Table S1). Accordingly, we previously showed that protein levels for multiple *H2a* genes are elevated in the DRG of Elongator knockouts in the context of normal transcript levels, suggesting that *H2a* genes may be regulated by Elongator in a coordinated fashion via their shared codon bias (Goffena et al., 2018).

In addition to elevated levels of the canonical *H2a* histones, protein levels of the variant histone *H2a.z* were also elevated in *Elp1* CKO embryos (Goffena et al., 2018). In fact, out of all the *H2a* histones, *H2a.z* showed the largest increase in expression (16-fold) (Table S1). Since reduced *H2a.z* is associated with over-proliferation and delayed differentiation of neural progenitors (Shen et al., 2018), we speculated that the opposite might also apply; that elevated expression of *H2a.z* in the CKO might contribute to the reduced progenitor proliferation and premature differentiation of TrkA+ neurons that occurs in the DRG. Since our proteome study identifying elevated H2A.Z levels was performed at E17.5, well after neural progenitors in the sensory ganglia have stopped dividing, we wanted to assess H2A.Z levels at E11.5 during the peak of neurogenesis. Prior to pursuing this question, we first sought to confirm the persistence of a robust phenotype in our model since our previous studies were conducted in 2013. Quantification of the number of neuronal progenitors (Pax3+) at E11.5 and the number of TrkA+ neurons at E12.5 confirm a significant reduction in both cell types as we previously demonstrated (Fig. S1).

**Fig. 1.**
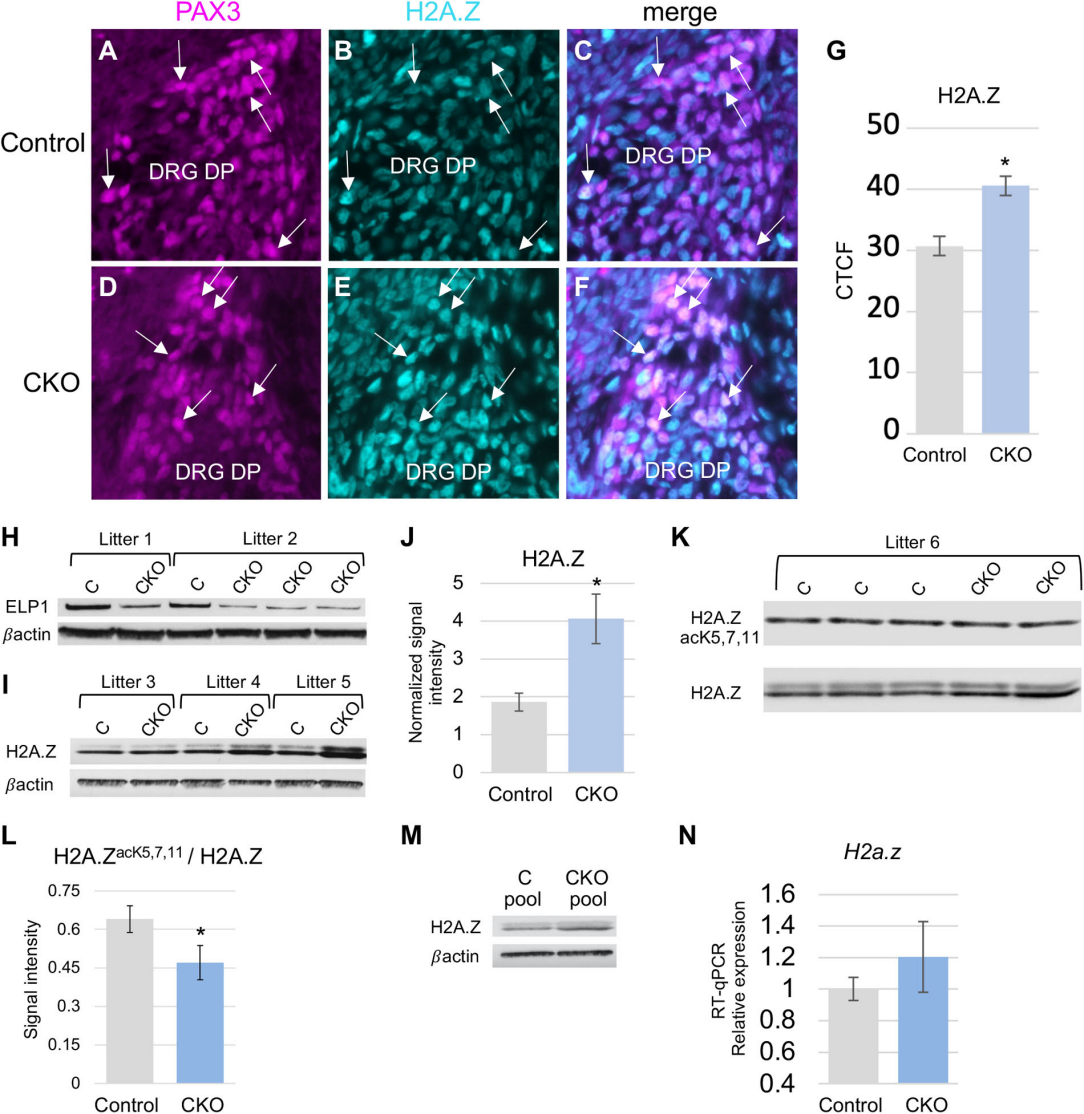
**H2A.Z levels are elevated in neuronal progenitors that are deficient for *Elp1*.** (A–G) E11.5. (A,D) PAX3 expression in dorsal pole DRG progenitors. (B,E) H2A.Z expression in the DRG. (C,F) PAX3/H2A.Z overlay. (G) Quantification of corrected total cell fluorescence (CTCF) shows that H2A.Z levels are elevated in PAX3+ cells in the DRG of *Elp1* CKOs (*P=*0.004). (H–L) E17.5. (H) ELP1 levels are diminished in the brains of *Wnt1-Cre*; *Elp1^LoxP^*^/*LoxP*^ CKO embryos. Residual ELP1 protein present in the CKO lanes is likely due to the presence of tissue outside of the *Wnt1-Cre* activity domain. (I) H2A.Z levels are consistently elevated in the brains of *Wnt1-Cre; Elp1^LoxP^*^/*LoxP*^ CKO embryos. (J) Average, normalized signal intensity=1.86 for the control and 4.06 for the CKO (*P*-value*=*0.04, *n*=3 control and 4 CKO). (K,L) Although H2A.Z levels are elevated in the absence of *Elp1*, the levels of acetylated H2A.Z are normal, resulting in a reduced ratio of acetylated to total H2A.Z in the CKO. (M) Adult *Pax6-Cre; Elp1^LoxP^*^/*LoxP*^ CKO mice exhibit elevated levels of H2A.Z in the retina. Average, normalized signal intensity in pooled samples=0.51 in the control and 0.91 in the CKO. The faint band migrating slightly above the H2A.Z band in I and K corresponds to the ubiquitinated form of H2A.Z (Lamaa et al., 2020). (N) Quantitative RTPCR shows that *H2a.z* transcript levels may be slightly elevated in *Elp1 CKO* embryos, but the difference is not significant (*P*=0.21, *n*=3 control and 3 CKO). Error bars denote SEM; scale bar: 20 µm. C, control; CKO, conditional knockout; DRG DP, dorsal root ganglion dorsal pole; CTCF, corrected total cell fluorescence.

Having verified our model, quantitative immunohistochemistry was performed to specifically assess H2A.Z levels in neural progenitors (PAX3+) during DRG neurogenesis (E11.5). PAX3 is a member of the paired-box family of transcription factors, and is expressed by neural progenitors, including those that give rise to the abundant small-diameter TrkA+ sensory population that is most severely impacted in both FD and our CKO mouse model (George et al., 2010, 2013; Pearson et al., 1978; Koblar et al., 1999; Underhill and Gros, 1997). As shown in Fig. 1, H2A.Z levels are significantly elevated in PAX3+ progenitor cells in the DRG of *Elp1* CKO embryos (Fig. 1A–G).

To determine whether elevated H2A.Z is specific to the DRG, or rather is more widespread, we also quantified H2A.Z in the brains of *Elp1* CKO and control embryos via western blot. Both *Elp1* and *Wnt1* are expressed in the developing brain at E17.5 (Fig. 1H) (https://developingmouse.brain-map.org/gene/show/22165), and accordingly, ELP1 levels in the CKO brain are decreased at this timepoint (Fig. 1H). In contrast, and like our findings in the DRG via immunohistochemistry, western blots show that H2A.Z levels are significantly elevated in the CKO brain (Fig. 1I,J). Since H2A.Z activity is regulated by its acetylation, we also quantified the ratio of acetylated H2A.Z to total H2A.Z and found it to be significantly diminished in the CKO (Fig. 1K,L). These data demonstrate that the level of total H2A.Z present in the cell, as well as the percentage of H2A.Z that is acetylated is significantly impacted by the loss of *Elp1*. Finally, to determine whether H2A.Z upregulation is unique to *Wnt1-Cre; Elp1^LoxP^*^/*LoxP*^ knockouts, we also quantified H2A.Z in pooled retinas from *Pax6-Cre*; *Elp1^LoxP^*^/*LoxP*^ mice that exhibit progressive retinal degeneration (Ueki et al., 2018). H2A.Z levels in these mice were again similarly elevated showing an approximate twofold increase in signal intensity (Fig. 1M), suggesting that H2A.Z upregulation may be a universal consequence of Elongator dysfunction.

Like the canonical *H2a* genes, the mRNA transcript for *H2a.z* is small, consisting of only 129 codons (Table S1). However, unlike the canonical *H2a* family members, *H2a.z* has a much higher AA:AG ratio (0.5 in mouse compared to an average of 0.08 for the canonical H2As) and this is in the context of a similar total number of lysine, glutamine, and glutamic acid codons. In fact, except for two members (*H2a.j* and *H2a.x*), the H2A variant histones all have AA:AG ratios above 0.33, with an average of 0.55 (Table S1). We previously showed that transcripts in this size range are much more likely to be upregulated in the absence of Elongator if their AA:AG ratios are significantly less than 0.3 (Goffena et al., 2018). Thus the majority of *H2a* variants in mouse, including *H2a.z*, are unlikely candidates for direct Elongator regulation (Goffena et al., 2018). Interestingly though, *H2a.z* is more significantly elevated in the context of *Elp1* loss than are the canonical *H2as* (Table S1). To gain insight into whether the misregulation of H2A.Z occurs at the transcript or protein level, quantitative RTPCR was used to examine *H2a.z* transcript levels. Although a trend toward a slight increase in relative expression was observed, the difference was not significant (Fig. 1N). Since SUMOylation and the ubiquitin proteosome system (UPS) are the primary regulators of H2A.Z levels (Takahashi et al., 2017), this result was not unexpected. Sirtuin1 has been shown to negatively regulate H2A.Z by targeting it for degradation via the UPS pathway (Chen et al., 2006). Since SIRT1 was not detected in our previous proteome study, we performed quantitative IHC in the DRG (not shown) and western blots in the brain (Fig. S2) and found normal levels of SIRT1 via both assays. However, multiple other enzymes involved in the UPS pathway are misregulated in the absence of *Elp1* (Table S2) including *UBR1*, an E3 ubiquitin ligase, which has been shown to decrease H2A.Z levels *in vivo* (Sharma et al., 2013). We found a 60-fold decrease in UBR1 in our previous proteome study in the context of normal transcript levels (Table S2). At over 1700 codons and an AA:AG ratio of 1.0, *Ubr1* is a strong candidate for diminished expression in the absence of Elongator (Goffena et al., 2018). Thus, although codon bias may not directly influence the translational efficiency of the *H2a.z* transcript, codon bias of downstream genes that determine H2A.Z longevity may have a larger impact on the overall levels of this histone variant within the cell.

### Notch target genes are misregulated in *Elp1* CKO embryos in the context of elevated histone H2A.Z

**Fig. 2.**
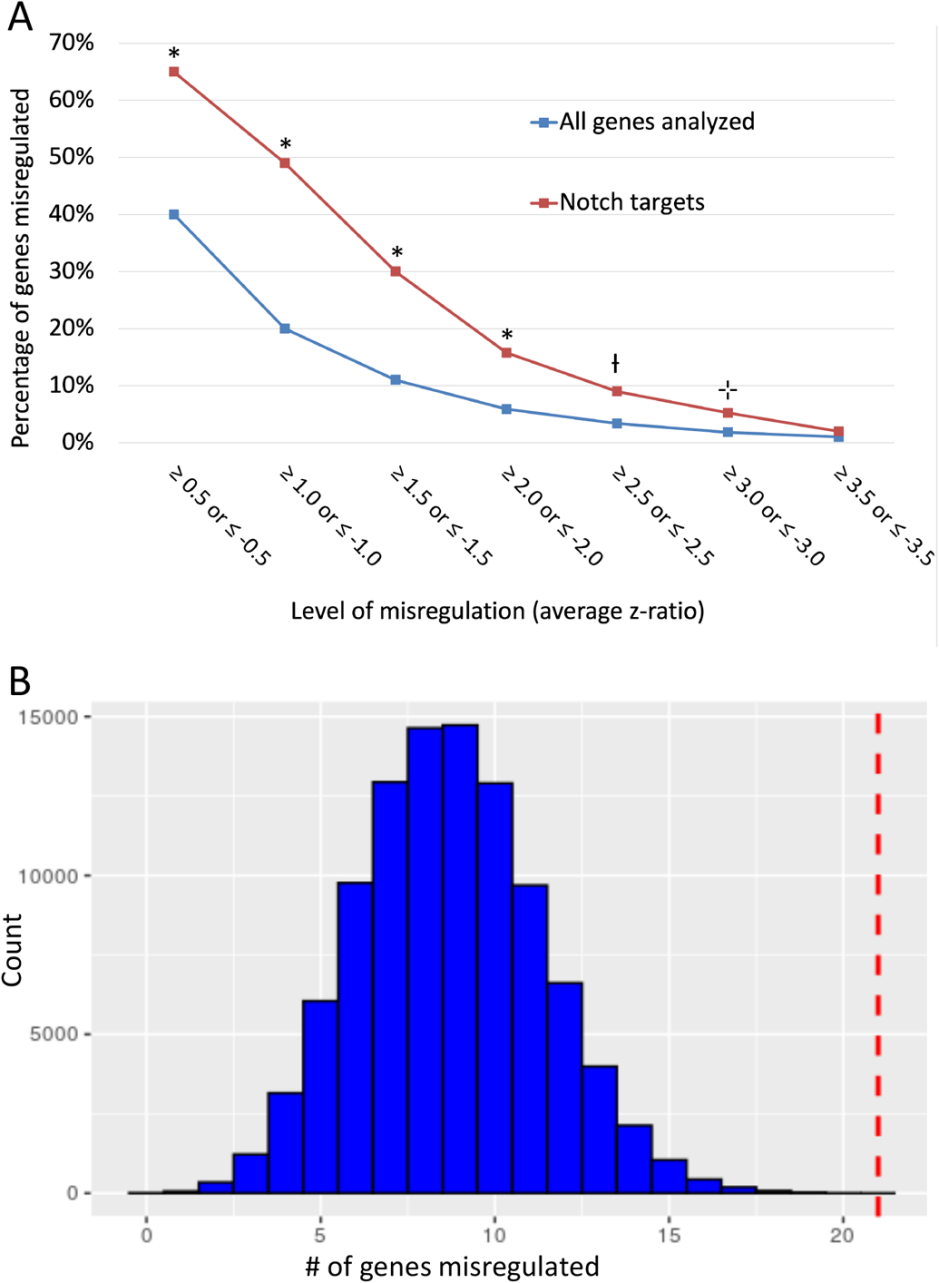
**Notch target genes are misregulated in *Elp1* CKO embryos.** (A) The percentage of Notch targets that are misregulated in *Elp1* CKO embryos is significantly higher than the background level of misregulation. * *P*≤0.000; † *P=*0.016; ⊹ *P=*0.086. (B) In a nonparametric randomization test using our data for a z-ratio ≥1.0 or ≤−1.0 and *P*=0.2 chance of misregulation, where a sample of 43 genes was randomized 100,000 times from a simulated transcriptome array, no events occurred where 21 genes (49%) or more were misregulated (dashed red line). Most samples landed around nine genes, corresponding to the 20% level of background misregulation observed at this z-ratio.

Notch signaling plays an essential role in achieving correct neuron numbers as well as correct cell type identity during the development of the nervous system and *Notch1* is robustly expressed by perimeter-localized progenitor cells in the DRG (Tsarovina et al., 2008). Previous studies have shown that H2A.Z directs the canonical Notch signaling pathway; H2A.Z deposition prevents Notch-directed cell proliferation by regulating the expression of Notch target genes, however, once acetylated, H2A.Z promotes proliferation again via regulation of the Notch pathway (Giaimo et al., 2018). Given the elevated levels of H2A.Z in our CKOs, we hypothesized that Notch target genes might also be misregulated. To test this hypothesis, we mined our previously published transcriptome data. As shown in Fig. 2A, Notch targets are significantly more misregulated than the overall level of misregulation observed in the CKO. 20% of all genes included in the transcriptome array (5201 out of 25,697) are misregulated at a z-ratio ≥1.0 or ≤−1.0. In contrast, 49% of Notch target genes (21 out of 43) are misregulated at this same level. Proportion hypothesis testing with a null hypothesis of 20% and alternative hypothesis that Notch target genes exhibit a larger than 20% level of misregulation, when applied to the observed 49% exhibited by Notch target genes (*n*=43), yields a *P*-value <0.000 allowing us to say with confidence that this difference is not likely due to chance alone. In fact, in a nonparametric randomization test where a transcriptome array was simulated with 5201 out of 25,697 genes misregulated (i.e. assuming a null hypothesis of approximately 20%), a sample size of 43 was taken from this simulated array 100,000 times, and there were no events where 49% of 43 genes (21 genes) or more were misregulated (Fig. 2B). This test further supports our claim that the increased level of misregulation observed in Notch target genes is due to factors beyond chance and may be a consequence of elevated H2A.Z.

### Trichostatin A rescues the number of TrkA DRG neurons *in vivo* and corrects misexpression of the Notch target gene, *Notch3*

**Fig. 3.**
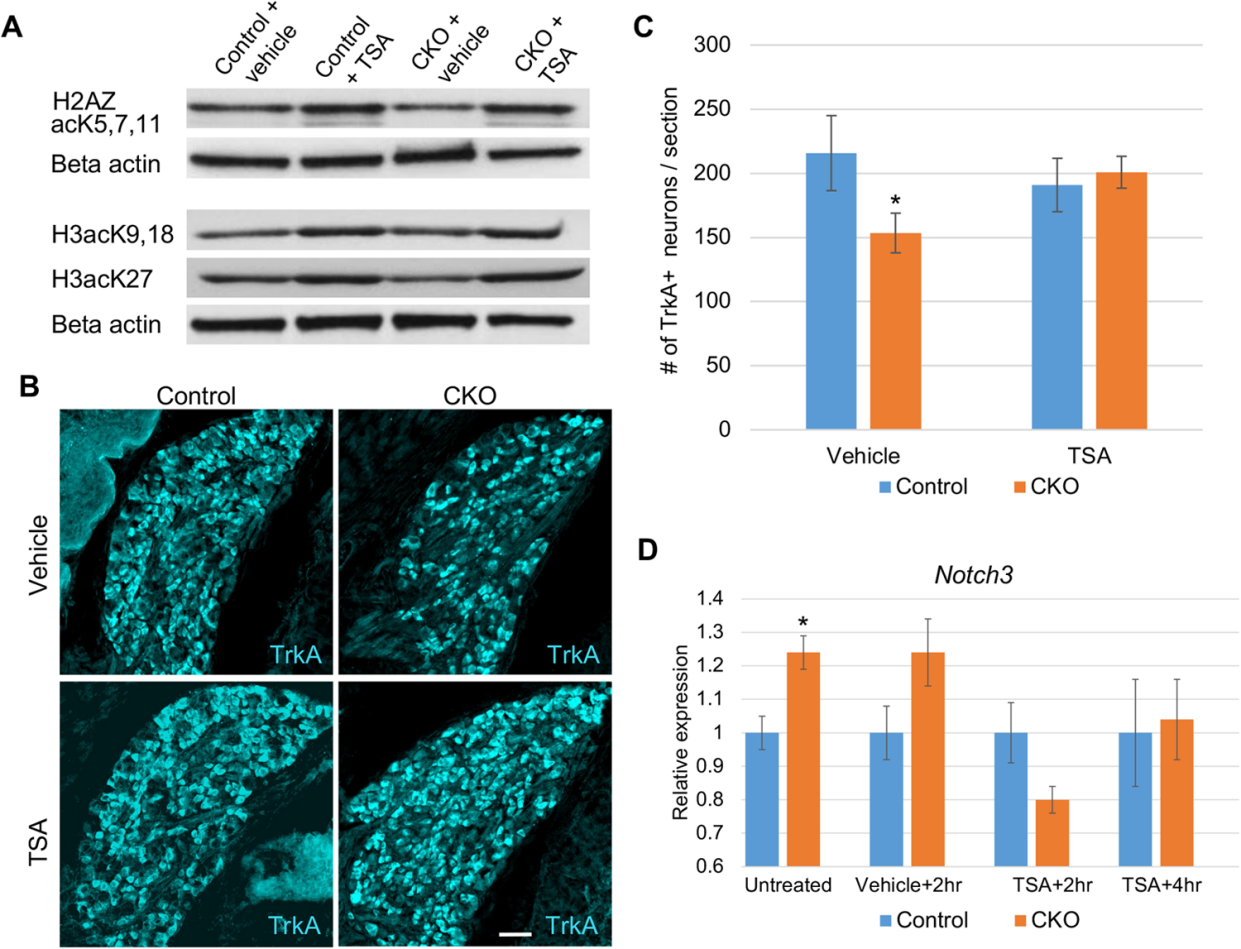
**Trichostatin A rescues the number of TrkA+ DRG neurons *in vivo*.** (A) Brains from pooled E11.5 embryos show elevated levels of acetylated H2A.Z and other histones 1.75 h after treating pregnant dams with TSA. Two brains were pooled per genotype and treatment. (B,C) E17.5. Three doses of TSA to pregnant dams during neurogenesis (E8.5, E10.5, and E12.5) returns the number of E17.5 TrkA+ neurons found in CKO embryos to the same average as that found in controls (*P*=0.52). *P*-value for vehicle treated control and CKO embryos=0.009. (D) *Notch3* expression levels diminish in response to TSA. E11.5 embryos were collected from pregnant dams that received either no injection (untreated), vehicle control, or TSA. Error bars denote SD; scale bar: 50 µm.

Since acetylation of H2A.Z promotes Notch-directed proliferation (Giaimo et al., 2018), we sought to determine whether histone deacetylase inhibitors that mediate histone hyperacetylation and target H2A.Z might counteract the effects of elevated H2A.Z per se, and potentially rescue compromised progenitor proliferation in our CKOs. Trichostatin A (TSA) is a potent histone deacetylase (HDAC) inhibitor that specifically inhibits class 1 and class 2 HDACs and includes H2A.Z as a target (Yoshida and Horinouchi, 1999; Bellucci et al., 2013; Narkaj et al., 2018). As shown in Fig. 3, administration of TSA to pregnant dams via intraperitoneal injection elevates acetylation of H2A.Z, as well as other embryonic histones.

To determine whether TSA treatment during neurogenesis would impact the reduced number of sensory neurons observed in our CKO mice, pregnant dams were given IP injections at three time points: E8.5, E10.5, and E12.5. We selected these timepoints as they span both the timeframe during which early TrkA+ neurons are born, and when late TrkA+ neural progenitors proliferate and their daughter cells finally differentiate (Farinas et al., 1996, 1998; Lawson and Biscoe, 1979). As shown in Fig. 3, treating CKO embryos with TSA during this window fully rescues TrkA+ afferents such that knockout and control embryos have comparable neuron numbers just prior to birth (Fig. 3B,C). This finding strongly supports the thesis that a major contributor to the reduced number of TrkA+ neurons in *Elp1* CKO embryos at E17.5 (George et al., 2013) and at birth (Jackson et al., 2014) is the loss of progenitors that give rise to this neuronal subset.

To determine whether the rescue effect of TSA could be attributed, at least in part, to an impact on Notch target gene expression, we analyzed the transcript levels of *Hes1* and *Notch3* with and without TSA. *Hes1* and *Notch3* are two well-known Notch targets that showed relatively high levels of misexpression in our previous transcriptome analysis at E17.5 (Table S3) (Goffena et al., 2018). At E11.5, pregnant dams received either no injection, injection of vehicle (25% DMSO in PBS), or injection of TSA (1 mg/kg in 25% DMSO in PBS). In mice that received TSA, embryos were collected at either 2- or 4-h post-injection. Embryonic neural tubes with attached DRG were then collected, RNA isolated, and cDNA synthesized. Although *Hes1* expression was normal in the CKO at E11.5 and its expression did not change in response to TSA (Fig. S3), *Notch 3* expression was elevated in the CKO (as it was at E17.5) and its expression diminished significantly in response to TSA (Fig. 3D). These results suggest that the expression levels of at least some Notch target genes respond to TSA administration, as would be expected given that H2A.Z acetylation increases in response to TSA and that H2A.Z acetylation is known to regulate the Notch signaling pathway (Giaimo et al., 2018). Notably, unlike *Notch1* whose expression corresponds with increased cellular proliferation, *Notch3* expression is associated with neuronal differentiation (Rusanescu and Mao, 2014). Thus, elevated levels of *Notch3* in the CKO may contribute to precocious differentiation and reduced numbers of sensory neurons (George et al., 2013). Accordingly, the ability of TSA to reduce *Notch3* expression may mediate, at least in part, its rescue effect on neuron number.

### Histone acetylation is normal in *Wnt1-Cre*; *Elp1^LoxP^*^/*LoxP*^ embryos

Previous *in vitro* studies in both lower organisms and humans have shown acetyltransferase activity for Elongator with histone H3 a preferred target over histone H4, and H3K14 being a specific target residue (Winkler et al., 2002; Wittschieben et al., 1999, 2000; Hawkes et al., 2002). Since histone acetylation is known to impact chromatin structure and accessibility, we wanted to investigate whether TSA's ability to rescue neurogenesis might be mediated in part via simply counteracting a histone acetylation deficit that could cause inappropriate condensation of chromatin in the absence of *Elp1*. To test this hypothesis, we quantified the acetylation levels of histone H3K14 in CKO embryos versus controls. Although the absence of a robust antibody for IHC precluded quantification in the DRG, western blots show similar acetylation levels for this histone in the brains of E17.5 CKOs and controls (Fig. S4A,B). We also quantified acetylation levels of H3K9,18, and again found no differences between the CKO and controls (Fig. S4C,D).

### Obvious alterations in chromatin structure are not evident in *Elp1* CKO embryos

**Fig. 4.**
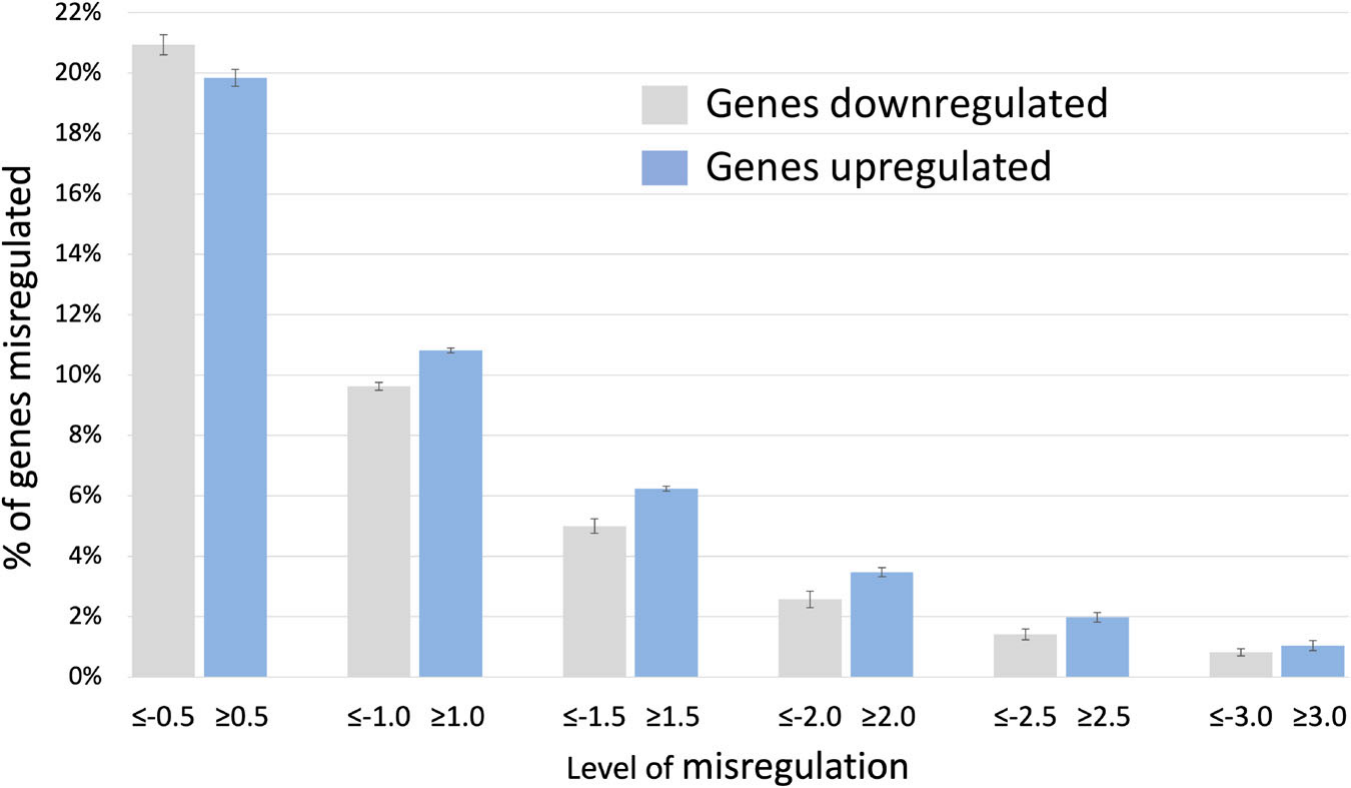
**Elevated H2A histones in *Elp1* CKO embryos do not disproportionately suppress gene expression.** The percentage of genes that are downregulated in *Elp1* CKO embryos is similar to the percentage that are upregulated. In fact, at higher levels of misregulation, more genes appear to be upregulated than suppressed.

**Fig. 5.**
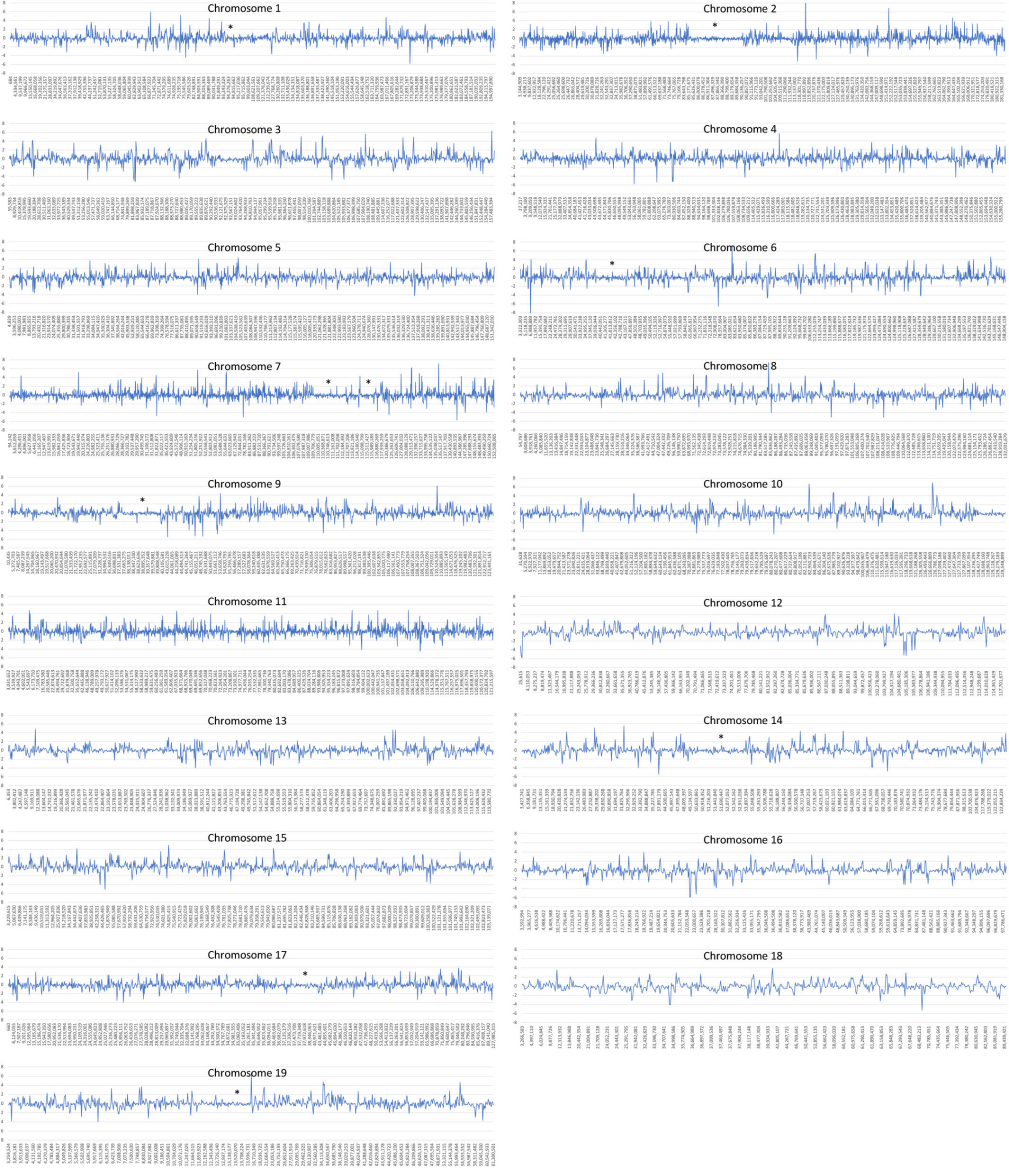
**Elevated H2A histones do not significantly alter chromatin structure in *Elp1* CKO embryos.** Average z-scores for all genes included in the transcriptome analysis of *Wnt1-Cre; Elp1^LoxP^*^/*LoxP*^ embryos were plotted according to their chromosomal location. Significant stretches where multiple adjacent genes were downregulated, indicating the formation of heterochromatin, were not detected. In contrast, normally occurring regions of heterochromatin surrounding *Olfr* genes are apparent (indicated by asterisks above chromosomal segments where both up and down misregulation are reduced comparted to neighboring regions). *Chromosome 1: approximate *Olfr* gene location: bp 94,376,000–94,517,000; *Chromosome 2: 85,170,000–90,136,000; *Chromosome 6: 42,410,000–43,186,000; *Chromosome 7: 109,625,000–112,518,000; 113,821,000–114,292,000; 114,959,000–16,0037000; *Chromosome 9: 37,519,000–39,995,000; *Chromosome 14: 50,514,000–53,118,000; *Chromosome 17: 37,337,000–38,473,000; *Chromosome 19: 12,749,000–13,994,000.

In the absence of histone acetylation differences, we next considered whether the elevated levels of H2A histones themselves in the CKOs could still be altering chromatin structure. Multiple H2A histones are elevated in *Elp1* CKO embryos (Goffena et al., 2018). Since the overexpression of histones has been shown to condense chromatin and block transcription *in vitro* (Singh et al., 2010; Steger and Workman, 1999), we considered whether TSA, via hyperacetylating histones throughout the nucleus, might loosen inappropriately condensed regions of DNA present in the CKO where transcription would otherwise be suppressed. To explore this possibility, we again mined our previously published transcriptome data. If elevated H2A histones are indeed compacting DNA in the absence of *Elp1*, we would expect to see an overall decrease in gene expression. However, our transcriptome data show that overall, the number of downregulated genes is roughly equal to the number of upregulated genes. As shown in Fig. 4, approximately 21% of genes are downregulated at an average z-ratio ≤−0.5 and 20% of genes are upregulated at a z-fold level ≥0.5. Interestingly, at all higher levels of misregulation, slightly more genes are upregulated than downregulated (Figs 4 and 5). Additionally, we did not detect regions within the genome that are inappropriately silenced. Fig. 5 and Table S4 show a graphical representation of altered gene expression across each chromosome. Significant stretches where multiple adjacent genes are downregulated are not present. In contrast, normally occurring regions of heterochromatin corresponding to the odorant receptor *Olfr* genes are quite evident (asterisks in Fig. 5). In mouse there are ∼1000 *Olfr* genes that mediate specificity in olfaction. They occur in large clusters throughout the genome that are characterized by regions of both constitutive and facultative heterochromatin (Armelin-Correa et al., 2014; Clowney et al., 2012; Magklara et al., 2011). Fig. 5 shows that, as expected, both upregulation and downregulation are suppressed in these regions (asterisks) compared to the overall level of misregulation across the genome (also see Table S4 with chromosomal locations of the *Olfr* genes). Our detection of heterochromatin surrounding the *Olfr* genes shows that the resolution of our analysis was strong enough to identify changes in chromatin structure had they been present. These data indicate that although H2A histones are upregulated in *Elp1* CKOs, their level of upregulation or incorporation into the genome is not high enough to significantly alter chromatin structure. As such, the rescue effect of TSA is not likely mediated via counteracting regions of inappropriate heterochromatin present in *Elp1* CKOs.

## DISCUSSION

Here we show that the histone variant *H2a.z* and multiple Notch targets, genes that regulate both proliferation and differentiation of neural progenitors, are perturbed in the context of *Elp1* loss. Following the completion of neural crest cell migration, proliferation and differentiation are two cellular processes that play critical roles in the development of the nervous system. Compromised proliferation and/or precocious differentiation will not only lead to deficient neuron numbers but can also alter cell fate decisions since neuronal subtype identity is often timing dependent. On the other hand, unhindered proliferation has its own consequences including cancer. Notch signaling during the development of the nervous system plays a truly critical role in controlling these two activities (Bao and Cepko, 1997; Henrique et al., 1997; Austin et al., 1995; Silva et al., 2003), and interestingly, H2A.Z plays a supervisory role in the Notch signaling cascade; its incorporation prevents Notch-mediated progenitor proliferation, while its depletion increases proliferation (Giaimo et al., 2018). An additional layer in this regulatory scheme includes post-translational modifications of H2A.Z with acetylation acting as a switch that supports Notch-induced cell division (Giaimo et al., 2018).

Our previously published studies demonstrate that in the absence of Elongator, neural progenitors exit the cell cycle prematurely and either differentiate precociously into neurons or die through apoptosis, resulting in a depleted pool of progenitors such that the full complement of TrkA+ sensory neurons is never achieved (George et al., 2013). The present study complements our initial characterization of the *Wnt1-Cre; Elp1^LoxP^*^/*LoxP*^ CKO and provides insight into why PNS development is stunted in this model; our data show that perturbations in Notch signaling likely attenuate progenitor proliferation, a scenario that may contribute to reduced peripheral neuron numbers in FD children as well. Our current work conflicts with a 2014 report that also used a *Wnt1-Cre; Elp1^LoxP^*^/*LoxP*^ CKO mouse but failed to detect abnormal progenitor or neural numbers at E12.5 (Jackson et al., 2014). The authors maintain that the reduced number of neurons present later in development is due to the death of post mitotic neurons via failed innervation and nerve growth factor (NGF) signaling. In the present study we again quantified the number of Pax3+ DRG progenitors at E11.5 and TrkA+ neurons at E12.5 in *Wnt1-Cre*; *Elp1^LoxP^*^/*LoxP*^ embryos and found significantly reduced numbers of both, confirming our previously published data (George et al., 2013). Furthermore, we demonstrate that hyperacetylating histones exclusively during neurogenesis rescues the number of neurons just prior to birth. These results strongly indicate that the reduced number of neurons present in *Wnt1-Cre*; *Elp1^LoxP^*^/*LoxP*^ embryos at this later time point is at least in part due to a failure during neurogenesis rather than to an exclusive failure of innervation later in development. The discrepancies between our findings and those of Jackson and colleagues were likely due to a number of differences in experimental approach. Unlike Jackson et al., we separately quantified both mitotically active phosphorylated histone H3+ cells, as well as the number of Pax3+ neural progenitors at E11.5 throughout the entire trunk axial level (George et al., 2013). Counts by Jackson and colleagues were performed using Ki67, a marker that recognizes both precursor cells that have exited the cell cycle, as well as mitotically active progenitors (Jackson et al., 2014). Additionally, since their counts were performed at E12.5 at the level of the T1 vertebra, the authors were likely including glial precursors and progenitors; in vertebrates there is a rostral to caudal gradient in development and E12.5 primarily corresponds to gliogenesis at the T1 level while neurogenesis is nearly complete (Lawson and Biscoe, 1979). Finally, since the majority of DRG neurons lost in *Wnt1-Cre*; *Elp1^LoxP^*^/*LoxP*^ knockouts express TrkA, it is more likely that a deficiency in neuron number would have been detected had the authors quantified the TrkA+ number at E12.5, rather than exclusively counting total neurons. Further supporting a role for Elongator in neurogenesis, another more recent paper showed that loss of Elongator in cortical progenitors leads to microcephaly via premature differentiation of apical progenitors directly into neurons, as well as the loss of intermediate progenitors (Laguesse et al., 2015). The expression of *Elp1* in the ventricular zone of the spinal cord and brain and within the dorsal pole and perimeter progenitor zones of the DRG also support a function for Elongator in neurogenesis (Chaverra et al., 2017; George et al., 2013; Hunnicutt et al., 2012).

Here we show that three doses of the histone deacetylase inhibitor TSA during the time frame of neurogenesis fully rescues the number of TrkA+ sensory neurons that are present in the DRG of *Wnt1-Cre*; *Elp1^LoxP^*^/*LoxP*^ embryos just prior to birth. This rescue effect may have been mediated via multiple pathways. First, Elongator was previously thought to function as a histone acetyltransferase. If histone acetylation were indeed directly compromised in the absence of Elongator, the use of an HDAC inhibitor might simply counteract depleted levels of acetylated histones. Our data showing normal histone acetylation in *Elp1* CKOs, however, contradict such a model. Alternatively, we previously showed that multiple AG-biased canonical H2A histones are upregulated in the absence of *Elp1* (Goffena et al., 2018), potentially altering chromatin structure. By hyperacetylating histones throughout the nucleus, TSA may have loosened inappropriately condensed regions of DNA, helping to restore normal transcript levels. The transcriptome analyses performed in this study, however, indicate that in fact significant alterations in chromatin structure are not likely present in *Elp1* depleted cells, contradicting a model where HDAC inhibition rescues via releasing inappropriate heterochromatin. Finally, a specific impact on the histone variant H2A.Z and a downstream effect on Notch target genes may have mediated TSA's rescue effects. Here we show that H2A.Z levels are significantly elevated in neural progenitor cells in the absence of *Elp1* and that Notch signaling is also perturbed as a likely consequence. Additionally, we show that H2A.Z is in fact hyperacetylated by TSA and furthermore, that TSA corrects elevated expression of the Notch target gene *Notch3*. Since *Notch3* promotes neural differentiation at the expense of progenitor proliferation (Rusanescu and Mao, 2014), this effect of TSA, as well as a likely impact on other Notch target genes, may mediate TSA's ability to rescue sensory neuron number in *Elp1* CKO embryos. Additionally, previous studies have shown that TSA not only impacts H2A.Z acetylation, but also decreases H2A.Z protein levels while increasing SIRT1, a negative regulator of H2A.Z (Baptista et al., 2013). As such, the combined effects of TSA likely decrease overall H2A.Z levels and increase the ratio of acetylated H2A.Z to total H2A.Z, both of which promote Notch directed proliferation.

Separate from its role in the canonical Notch pathway, H2A.Z also plays a critical role during mitosis, with increased H2A.Z mediating sister chromatid cohesion during metaphase and decreases in H2A.Z facilitating chromatid separation during anaphase (Martins-Taylor et al., 2011; Sharma et al., 2013). Inappropriately high levels of H2A.Z could thus lead to alterations in chromosome formation/separation, genome instability, and progenitor cell death. Our previous findings that neural progenitors die in *Elp1* CKO embryos via p53-mediated apoptosis, as well as elevated levels of DNA damage, support this possibility (George et al., 2013; Goffena et al., 2018). Overall, our data support a model wherein the observed rescue effect of TSA is mediated via counteracting the negative effects of elevated H2A.Z present in the absence of *Elp1*.

Through the addition of tRNA U34 modifications, Elongator tunes the translation rate and hence the protein levels of genes that preferentially use either AA- or AG-ending codons for lysine, glutamine and glutamic acid. Since *H2a.z* is not significantly codon-biased, its increased expression in *Elp1* CKOs is not likely due to an elevated translation rate of the *H2a.z* transcript. Although a comprehensive dissection of the pathways that do mediate *H2a.z* elevation are beyond the scope of this study, we present data indicating that misregulation of codon-biased genes involved in the UPS pathway may be the culprit. Thus, although Elongator and codon usage may fine tune protein levels, the downstream effects of mistuning depend on the type of gene mis-tuned, with perturbations of genes involved in pathways like the ubiquitin degradation pathway having large downstream effects.

In summary, the present study demonstrates that both *H2a.z* and the Notch signaling pathway, both of which are key regulators of neuronal progenitor proliferation and differentiation, are significantly perturbed in *Elp1* CKOs. The misregulation of these genes almost certainly contributes to the stunted development of the peripheral nervous system that is a hallmark feature of *Elp1* loss. Furthermore, the ability of the histone deacetylase inhibitor TSA to impact Notch signaling through acetylation of H2A.Z likely explains the molecular mechanism via which TSA rescues PNS development in *Elp1* CKO embryos.

## MATERIALS AND METHODS

### Immunohistochemistry (IHC)

All washing, blocking, secondary antibody, and postfixation steps were performed at room temperature. All other steps were performed at 4°C unless stated otherwise. Embryos were fixed in 4% paraformaldehyde/phosphate-buffered saline (PBS) for 20 min (E11.5-12.5) or 2 h (E17.5), rinsed in PBS, cryoprotected through a series of sucrose solutions in PBS (15%, 30%), incubated for 2 h in a 1 : 1 mixture of 30% sucrose and optimal cutting temperature (OCT) compound (Tissue-Tek, Torrance, CA, USA), followed by 2 h in OCT. Embryos were then frozen and cryosectioned at 16 µm. For immunostaining, slides were bathed in TBS (tris-buffered saline) for 10 min, followed by NGS block (10% normal goat serum, 1% glycine, 0.4% Triton X-100 in 30 mM Tris, 150 mM NaCl) for 1 h, and overnight incubation in primary antibody (in NGS block). Slides were then rinsed in NGS block, incubated in Alexa Fluor secondary antibody (1:2000 in NGS block) for 1 h, rinsed in 3 : 1 TBS:NGS block, and mounted in Prolong Antifade Diamond (Invitrogen, La Jolla, CA, USA). Control and experimental embryos were cryosectioned on the same day and sections were typically incubated in primary antibody on the same day that they were sectioned. Microscopy images were captured using a Nikon TE200 inverted microscope and were captured with a QImaging QICAM 12-bit Mono Fast 1394 Cooled camera and QCapture software. Identical exposure times, gain, and offset settings were used to capture control and experimental images. Primary antibodies included the following: PAX3 (Developmental Studies Hybridoma Bank, 2 µg/ml), TRKA (Louis F. Reichardt, University of California, San Francisco, CA, USA, 1:1000), H2A.Z (Proteintech, 16441-1-AP, 1:100).

### Cell counting and analysis for IHC

For E17.5 cryosectioning and TRKA counts, 16 µm sections were collected from the mid-lumbar axial level (six total slides with 20 sections per slide), typically containing sections from six to eight DRG. The center-most section of each of six DRG (three from the left half of the body and three from the right half of the body) was identified, and the two sections flanking that center section were imaged for a total of 12 sections per embryo. Manual counts were performed blind. For counts of E12.5, and E11.5, center sections from ganglia throughout the entire length of the trunk from forelimb to hind limb were manually counted blind. In all cases, identical gain and offset parameters were used for control and experimental slides and a minimum of three control and three CKO embryos were analyzed. Corrected total cell fluorescence (CTCF) was measured using ImageJ and the ten brightest cells per center section were averaged. Data are presented as the average CTCF±SEM. Statistical significance was determined by an unpaired Student's *t*-test.

### Quantitative real-time PCR

Quantitative real-time PCR (qRTPCR) for *H2a.z* was performed using the Viia-7 qPCR System by Applied Biosystems and TaqMan Gene Expression Assays: Mm05916395 (*H2a.z*), Mm01342805 (*Hes1*), Mm01345646 (*Notch3*) (Thermo Fisher Scientific). Relative expression levels or fold change are shown normalized against Gapdh (TaqMan Gene Expression Assay: Mm99999915). For *H2a.z*, DRG were collected from three E17.5 control (*Wnt1-Cre*; *Elp1^+^*^/*LoxP*^) and three CKO (*Wnt1-Cre*; *Elp1 ^LoxP^*^/*LoxP*^) embryos. For *Hes1* and *Notch3*, the neural tube and attached DRG were collected from three E11.5 control and CKO embryos (genotypes as above). RNA was extracted using the Qiagen RNeasy Mini Kit and cDNA synthesized via the High-Capacity RNA-to-cDNA Kit (Applied Biosystems). Each reaction was performed in triplicate. Bars represent SD calculated from three Delta Delta Ct Expression values for each genotype.

### Western blots

Tissue was prepared by homogenization in radioimmunoprecipitation assay (RIPA) buffer (150 mM NaCl, 1% Nonidet P-40, 0.5% sodium deoxycholate, 0.1% SDS, 25 mM Tris pH 7.4) with protease and phosphatase inhibitors (Cell Signaling Technology) or histone extraction using a histone extraction kit (EpiGentek Group INC.). Protein concentrations of all samples were determined using biocinchoninic acid (BCA) protein assay (Thermo Fisher Scientific). Equal amounts of protein were incubated with Bolt 4x LDS Sample Buffer and 10× Sample Reducing Agent at 70°C for 10 min and then run on a Bolt 4-12% Bis-Tris Plus gel (Thermo Fisher Scientific). Gels were run at 110 V for 60–80 min depending on protein molecular weight. Gels were removed, equilibrated in 20% ETOH for 10 min, rinsed and protein transferred using the iBlot 2 Transfer Device (Thermo Fisher Scientific) to a nitrocellulose membrane. Program specificity was determined by molecular weight of proteins transferred. Membranes were then probed using diluted primary and secondary antibodies via the iBind Flex Device (Thermo Fisher Scientific) and incubated for 3 h to overnight. Primary antibodies: ELP1 (1:800; Abnova #PAB12857), H2A.Z (1:2000; Proteintech #16441-1-AP), H2A.Z acK5,7,11 (1:2500; EMD Millipore, #ABE1363), H3acK27 (1:10,000; Abcam, #ab4729), H3acK14 (1:1000; RevMAb Biosciences, #31-1032-00), H3acK9,18 (1:2500; EMD Millipore, #07-593), Sirt1 (1:2000; Proteintech, #13161-1-AP). Beta Actin (1:10,000; Proteintech, #60008-1-Ig) was used as a loading control. Proteins were visualized using Goat anti-Rabbit Horseradish Peroxidase (HRP) and goat anti-mouse HRP secondary antibodies; 40 ng/ml or 10 ng/ml, respectively. Blots were then washed and placed in 1 : 1 ratio; 10 ml total, of PLUS Chemiluminescent Substrate (Thermo Fisher Scientific) and agitated for 5 min. Blots were developed on B100 Blue X-ray film (VWR). Films were visualized and imaged using transilluminator and protein bands were quantified with ImageStudioLite. For Figures 1H-L the hindbrain/midbrain region was isolated from E17.5 embryos. For Fig. 1M, the retina and optic nerves from three adult mice (3 months of age) per genotype were pooled. For Fig. 3A, whole brains from two E11.5 embryos were pooled. Protein bands were normalized against beta actin and analyzed via simple mathematics and unpaired Student's *t*-tests.

### Transcriptome and proteome data

For detailed methods see Goffena et al. (2018). Transcriptome data are available at Gene Expression Omnibus (www.ncbi.nlm.nih.gov/geo/) (reference GSE80130). Proteome data are available at ProteomeXchange (www.proteomexchange.org/) (reference PXD007869).

### Mice

The generation of *Wnt1-Cre; Elp1^LoxP^*^/*LoxP*^ CKO embryos has been previously described (George et al., 2013). Wnt1-Cre mice were purchased from the Jackson Laboratory (stock number 003829) and all strains were maintained on a C57BL/6 J background. *Wnt1-Cre*; *Elp1^LoxP^*^/*LoxP*^ embryos were used as experimental and both *Wnt1-Cre*; *Elp1^+^*^/*LoxP*^ and *Elp1^+^*^/*LoxP*^ littermates were used as controls. All genotyping was performed via routine PCR. *Elp1* CKO and wild-type alleles were distinguished using the following primers: forward (F), 5′-GCACCTTCACTCCTCAGCAT-3′ and reverse (R), 5′-AGTAGGGCCAGGAGAGAACC-3′. The presence of the *Wnt1-Cre* allele was detected using the following primers: F, 5′-GCCAATCTATCTGTGACGGC-3′ and R, 5′-CCTCTATCGAACAAGCATGCG-3′. All experiments were performed according to the National Institutes of Health Guide for Care and Use of Laboratory Animals and protocols were approved by the Montana State University Institutional Animal Care and Use Committee.

### TSA injections

Pregnant dams received intraperitoneal (IP) injections of freshly prepared Trichostatin A (Selleck Chemical, #S1045) dissolved in 25% DMSO, 75% sterile PBS at a concentration of 1 mg/kg of body weight on days E8.5, E10.5, and E12.5. To analyze the effect of Trichostatin A (TSA) on histone acetylation, pregnant dams received an IP injection as above on day E11.5 and embryos were collected 1.75 h later and genotyped. Whole brains from these embryos were pooled according to genotype and treatment and used for histone isolation and western blots (see above).

## Supplementary Material

Supplementary information
