# Supplementary material for: Loss of Elp1 perturbs histone H2A.Z and the Notch signaling pathway
Source: Biol Open. 2021 Sep 30;10(9):bio058979. doi: 10.1242/bio.058979 (PMC8496692; doi:10.1242/bio.058979)
Supplement: Supplementary information [file biolopen-10-058979-s1.pdf]

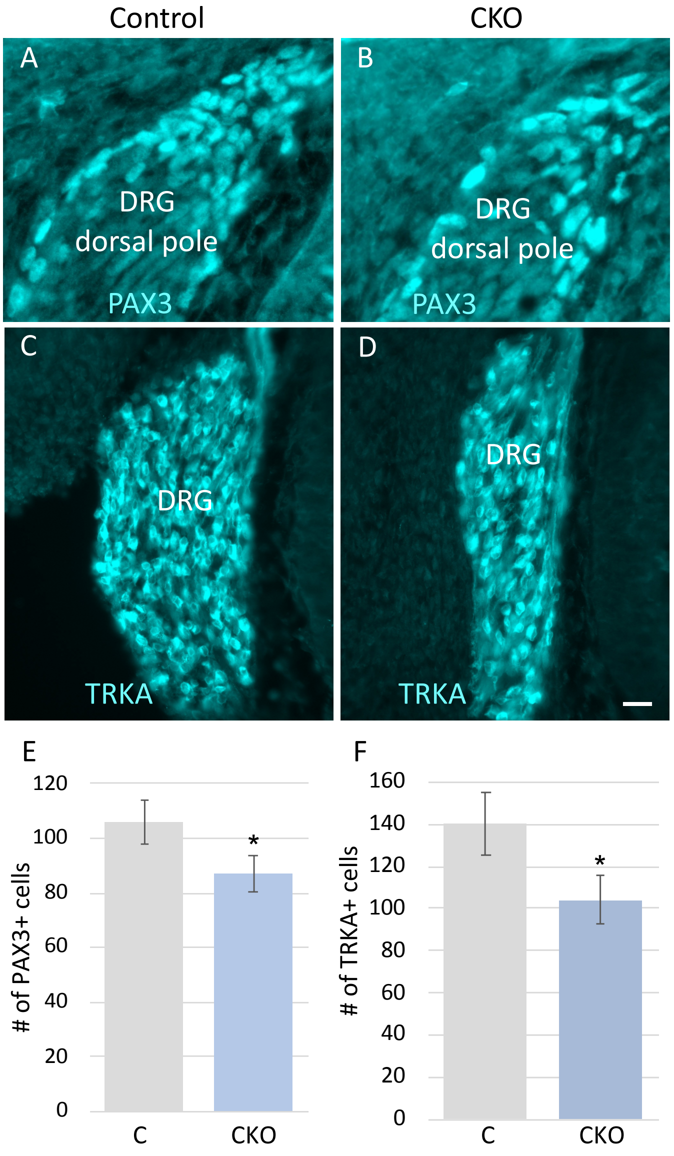

Fig. S1. Pax3+ progenitors and TrkA+ neurons are depleted in *Wnt1-Cre; Elp1* $^{LoxP/LoxP}$ CKO embryos during development of the dorsal root ganglia. A,B,E) E11.5. Pax3+ cells are significantly reduced in CKO embryos compared to controls. **P value* < 0.00, n = 3 control and 3 CKO. C,D,F) E12.5. TrkA+ neurons are significantly reduced in CKO embryos compared to controls. **P value* < 0.00, n = 3 control and 3 CKO. Scale bar = 25 μm; error bars denote SEM. CKO, conditional knockout; DRG, dorsal root ganglion.

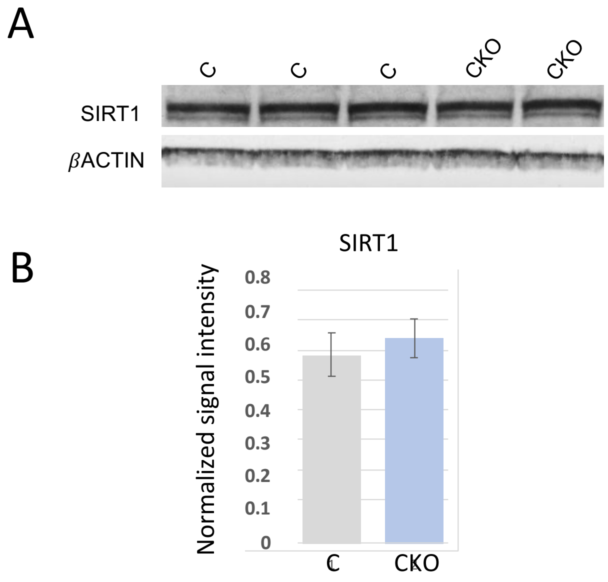

Fig. S2. Sirtuin1 levels are normal in *Elp1* CKO embryos.

A) E17.5. Western blots show that SIRT1 levels are the same in brain tissue from *Wnt1-Cre; Elp1* $^{LoxP/LoxP}$ CKO embryos and controls. B) Quantification of normalized band intensities from multiple litters. *P value* = 0.67, n = 4 control and 3 CKO.

Biology Open • Supplementary information

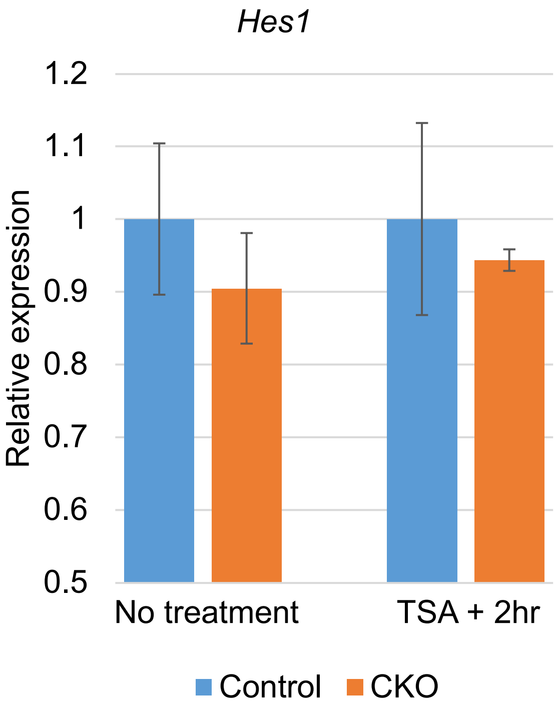

Fig. S3. *Hes1* transcript levels are normal in *Elp1* CKO embryos at E11.5 and are not impacted by TSA. Quantitative RTPCR shows that transcript levels of *Hes1* are normal in *Elp1* CKO embryos at E11.5 (*P* = 0.45, n = 3 control and 3 CKO) and that *Hes1* expression is not impacted by TSA (*P* = 0.53).

Biology Open • Supplementary information

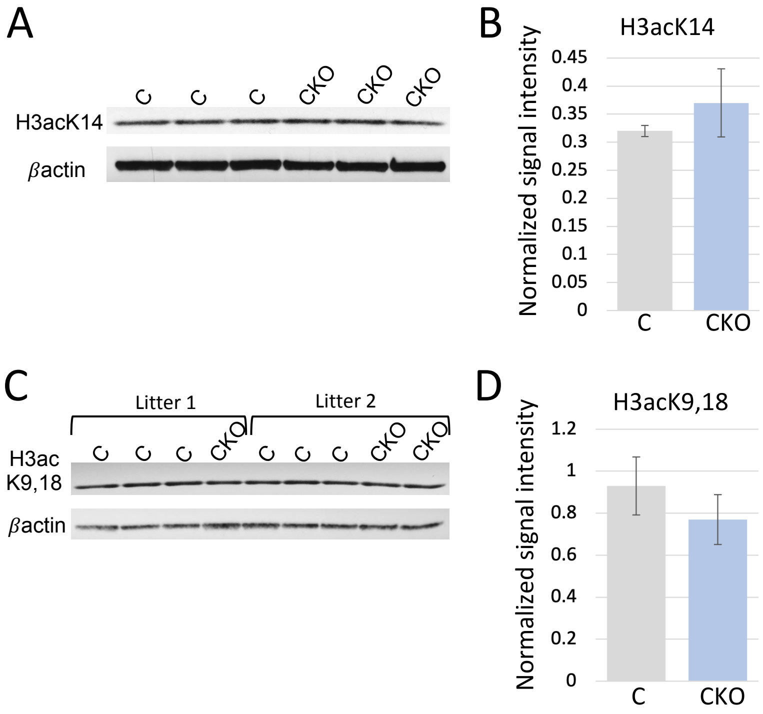

Fig. S4. Histone acetylation is normal in *Elp1* CKO embryos. E17.5. A) Western blots show that levels of acetylated H3acK14 are the same in brain tissue from *Elp1* CKOs and controls. B) Quantification of normalized band intensities. *P* = 0.49, n=3 control and 3 CKOs from the same litter. C) Western blots show that levels of acetylated H3acK9,18 are the same in brain tissue from *Elp1* CKOs and controls. D) Quantification of normalized band intensities. *P* = 0.14, n= 6 control and 3 CKOs from two separate litters.

Biology Open • Supplementary information

Table S1. H2A Histones *(Mus musculus)*

Click here to download Table S1

Table S2. Mouse ubiquitin proteosome system genes that are misregulated in the absence of Elp1

Click here to download Table S2

Table S3. Transcriptome results for Notch target genes (defined as genes whose expression levels have been demonstrated to change as a result of Notch signalling) (Giaimo et al., 2018; Borggrefe & Oswald, 2009).

Click here to download Table S3

Table S4. Transcriptome z-ratio values plotted against chromosome location. Scroll right for individual z-ratios.

Click here to download Table S4
